# Novel Therapies for the Treatment of Drug-Induced Liver Injury: A Systematic Review

**DOI:** 10.3389/fphar.2021.785790

**Published:** 2022-02-02

**Authors:** Mirjana Stanić Benić, Lana Nežić, Vesna Vujić-Aleksić, Liliana Mititelu-Tartau

**Affiliations:** ^1^ Department of Clinical Pharmacology, Clinical Hospital Centre Rijeka, Rijeka, Croatia; ^2^ Department of Pharmacology, Toxicology and Clinical Pharmacology, Faculty of Medicine, University of Banja Luka, Banja Luka, Bosnia and Herzegovina; ^3^ The Republic of Srpska Agency for Certification, Accreditation and Quality Improvement in Health Care, Banja Luka, Bosnia and Herzegovina; ^4^ Grigore T. Popa, University of Medicine and Pharmacy, Iasi, Romania

**Keywords:** drug-induced liver injury, hepatotoxicity, treatment, therapy, toxic hepatitis

## Abstract

Many drugs with different mechanisms of action and indications available on the market today are capable of inducing hepatotoxicity. Drug-induced liver injury (DILI) has been a treatment challenge nowadays as it was in the past. We searched Medline (*via* PubMed), CENTRAL, Science Citation Index Expanded, clinical trials registries and databases of DILI and hepatotoxicity up to 2021 for novel therapies for the management of adult patients with DILI based on the combination of three main search terms: 1) treatment, 2) novel, and 3) drug-induced liver injury. The mechanism of action of novel therapies, the potential of their benefit in clinical settings, and adverse drug reactions related to novel therapies were extracted. Cochrane Risk of bias tool and Grading of Recommendations Assessment, Development and Evaluation (GRADE) assessment approach was involved in the assessment of the certainty of the evidence for primary outcomes of included studies. One thousand three hundred seventy-two articles were identified. Twenty-eight articles were included in the final analysis. Eight randomized controlled trials (RCTs) were detected and for six the available data were sufficient for analysis. In abstract form only we found six studies which were also anaylzed. Investigated agents included: bicyclol, calmangafodipir, cytisin amidophospate, fomepizole, livina-polyherbal preparation, magnesium isoglycyrrhizinate (MgIG), picroliv, plasma exchange, radix Paeoniae Rubra, and S-adenosylmethionine. The primary outcomes of included trials mainly included laboratory markers improvement. Based on the moderate-certainty evidence, more patients treated with MgIG experienced alanine aminotransferase (ALT) normalization compared to placebo. Low-certainty evidence suggests that bicyclol treatment leads to a reduction of ALT levels compared to phosphatidylcholine. For the remaining eight interventions, the certainty of the evidence for primary outcomes was assessed as very low and we are very uncertain in any estimate of effect. More effort should be involved to investigate the novel treatment of DILI. Well-designed RCTs with appropriate sample sizes, comparable groups and precise, not only surrogate outcomes are urgently welcome.

## Introduction

Drug-induced liver injury (DILI) is one of the most frequent causes of acute liver failure (ALF), and one of the main reasons for drug withdrawal from the market (European Association for the Study of the Liver, 2019). The pathogenesis of DILI involves genetic, metabolic, and immune factors. Despite difficulties in the classification, DILI can be divided into three categories that share some common features as well as differences (Hoofnagle and Björnsson, 2019). The first category, direct hepatotoxicity, is caused by an intrinsically toxic drug with onset within 1–5 days after administration, and is predictable, dose-dependent and reproducible in animal models. The second is idiosyncratic DILI (iDILI) which is mostly dose-independent (although a dose threshold of 50–100 mg/day is usually required), unpredictable, and exhibits a variable latency to onset. Furthermore, iDILI is subdivided into two subcategories: allergic, immune-mediated, and non-allergic, non-immune-mediated (Kuna et al., 2018; European Association for the Study of the Liver, 2019; Hoofnagle and Björnsson, 2019). Immune-mediated iDILI seems to be generated by antigen recognition-mediated by helper T cells, whereas non-immune iDILI was found to be associated with variants of human leukocyte antigens, suggesting that adaptive immune response might influence both variants of iDILI (Daly and Day, 2009; Kuna et al., 2018). The first, immune-mediated iDILI has shorter latency (1–6 weeks) and is characterized by the occurrence of fever, rash, autoantibodies, eosinophilia, and Stevens-Johnson syndrome while non-immune mediated iDILI lacks these features and has a longer latency (1 month–1 year) (Vincenzi et al., 2018). An indirect DILI emerges as the third category caused by the biological action of the offending drug rather than by its toxic or idiosyncratic properties. All DILI categories are manifested by distinctly different phenotypes such as acute hepatitis and enzyme elevations (hepatocellular, cholestatic, or mixed type), acute hepatic necrosis, chronic hepatitis, fatty liver, bland cholestasis, sinusoidal obstruction syndrome, and ALF (Hoofnagle and Björnsson, 2019).

Up to date, extensive research has contributed to an understanding of the pathophysiology and molecular pathology of DILI, which facilitated predictability of hepatotoxicity triggered by drugs including identification of the risk factors and clinical diagnosis of DILI (Stine and Lewis, 2016).

However, specific treatment of acute or chronic DILI has not changed significantly other than supportive therapy and limited numbers of antidotes. Due to the perceived severity of the further liver injury, early discontinuation of the implicated drug, once the diagnosis of DILI is suspected, is the common therapeutic approach. Current pharmacotherapeutic interventions of DILI include the use of several specific antidotes, immunosuppressants, and known hepatoprotective agents (Chalasani et al., 2014; Saliba and Samuel, 2015; Stine and Lewis, 2016). Orally or intravenously administered N-acetylcysteine (NAC) is the best-proven agent in the treatment of paracetamol (acetaminophen) overdose caused liver injury or in the prevention of ALF, hepatotoxicity induced by antituberculosis and other causative drugs (Lee et al., 2009; Baniasadi et al., 2010; Hu et al., 2015). High doses of corticosteroids have been empirically used in the treatment of liver injuries associated with drug-induced hypersensitivity syndromes (Avancini et al., 2015), drug-related eosinophilic systemic syndrome (Lee et al., 2013), or secondary to the use of programmed cell death protein 1 (PD-1) inhibitors (Larkin et al., 2015; Huffman et al., 2018; Shah et al., 2020). The therapeutic efficacy of ursodeoxycholic acid (UDCA) in cholestatic or mixed DILI is largely documented in observational studies and case reports, although some controlled clinical trials have failed to confirm its positive effect (Marino et al., 2001; Chalasani et al., 2014; Robles-Diaz et al., 2021). The most promising results indicated that UDCA ameliorates DILI or attenuates the risk of developing drug-induced vanishing bile duct syndrome in some patients (O’Brien et al., 1996). Administration of cholestyramine, a bile-acid binding resin, in severe cholestatic DILI contributes to the elimination of hepatotoxic drugs that undergo enterohepatic circulation, binds bile acids in the intestine, and interrupts their enterohepatic circulation by increasing their fecal excretion (Ast and Frishman, 1990; Wong et al., 2009).

A lot of pharmacologic active substances have been investigated as hepatoprotective agents *in vitro* and *in vivo*. Although well-known silymarin has shown therapeutic potential in animal models of DILI, there are still inconclusive results of its efficacy in the treatment and prevention of hepatotoxicity induced by antituberculosis drugs (Gu et al., 2015; Marjani et al., 2019). Numerous natural compounds such as quercetin, a polyphenolic flavonoid, chitosan, a marine polysaccharide made from crustaceans, alginate, a polysaccharide derived from brown algae, collagen of marine origin, and L-carnitine demonstrated hepatoprotective effect and improved liver function in paracetamol overdose and iDILI, respectively, due to anti-inflammatory, antioxidant and antiapoptotic properties (Santhosh et al., 2007; Lheureux and Hantson, 2009; Ozcelik et al., 2014; Han et al., 2015; Barros et al., 2017; Shteyer et al., 2019). Cytokine-based therapeutic approach has been less explored as a potential treatment of DILI, although a low dose of interleukin-2 showed promising effectiveness in preclinical immune-mediated DILI due to induction of regulatory T-cells and balance between pro- and anti-inflammatory cytokines (Kumar et al., 2021). Extensive experimental and clinical research highlighted a number of natural compounds, chemical substances, and biologics, with potential therapeutic efficacy on liver injuries induced by various drugs. These represent generous research directions in the development of the innovative therapeutic approaches, and base for the use of nanotechnology in the optimization of pharmaceutical forms of drugs, in order to limit their liver toxicity (Madrigal-Santillán et al., 2014; Domitrović and Potočnjak, 2016).

The main objective of this systematic review was to critically summarize and discuss potential pharmacologically active substances considered as novel therapies in the treatment of DILI with presumed beneficial outcomes in clinical practice.

## Materials and Methods

We aimed to systematically assess the investigated novel therapies for the management of adult patients with DILI. Potential of novel therapies in the treatment of patients with DILI, long-term effects (included both positive and negative), and related adverse drug reactions obtained from (pre)clinical studies or any other sources were extracted. Additionally, any other relevant and specific issues related to the targeted therapeutic agents were added (e.g., mechanism of action, primary and secondary endpoints of included studies, DILI causality assessment tools used in primary articles).

The following databases, clinical trials registers, and relevant websites were searched:

(1) MEDLINE (via PubMed; 1946 to August 2021);
(2) Science Citation Index Expanded (via Web of Knowledge; 1900 to August 2021);
(3) The Cochrane Central Register of Controlled Trials (CENTRAL) (The Cochrane Library current issue);
(4) ClinicalTrials.gov (http://clinicaltrials.gov/);
(5) World Health Organization (WHO) International Clinical Trials Registry Platform (ICTRP) search portal (http://apps.who.int/trialsearch/); and
(6) Websites: https://livertox.nih.gov/; https://dilin.org/; https://www.fda.gov/science-research/liver-toxicity-knowledge-base-ltkb/ltkb-benchmark-dataset; https://toxico.nibiohn.go.jp/english/; www.spanishdili.uma.es, https://www.ema.europa.eu

The search strategy included the combination of three main search terms: 1) treatment, 2) novel, and 3) drug-induced liver injury and is available in Table 1. It was limited to the published articles in English. Inclusion criteria for relevant articles were: an original article, a confirmed diagnosis of DILI or toxic hepatitis induced by drugs in adults, describing the investigation or use of novel substances or drugs for DILI or toxic hepatitis treatment, observational studies, case reports and series and clinical trials of novel treatments of DILI. We used a timeframe filter in database search from the beginning of reporting up to August 2021, as we aimed not to miss any investigated agent in DILI treatment, considered novel according to investigators’ opinion and including clinical trials results, off-label and experienced-based use. Exclusion criteria were: liver injury caused by any other causes, except drugs; therapies already known and used for DILI treatment such as NAC, silymarin, corticosteroids, UDCA, cholestyramine, liver transplantation; preventive treatment for DILI; DILI in the pediatric population, and preclinical research. Drug-induced liver injury was defined as a spectrum of clinical liver diseases ranging from mild biochemical abnormalities to ALF, caused by drugs, drug metabolites, herbal and dietary supplements, and chemicals from the environment (European Association for the Study of the Liver, 2019).

**TABLE 1:** Search strategy.

Concept 1	AND
treatment*(MeSH] OR	
therap*(MeSH] OR	
medicine*(MeSH] OR	
drug*(MeSH] OR	
agent*(MeSH]	
Concept 2	AND
New(tiab] OR	
Novel(MeSH]	
Concept 3	
drug induced liver injury, chronic(MeSH] OR	
drug induced liver injury(MeSH] OR	
drug induced liver disease(MeSH] OR	
liver dysfunction(MeSH] OR	
abnormalities, drug induced(MeSH] OR	
DILI(tiab] OR	
hepatotoxicit*(tiab] OR	
toxic hepatitis(tiab]	
Medline (*via* PubMed) search query	
#4 Search (#1 AND #2 AND #3)	
#3 Search (drug induced liver injury, chronic(MeSH] OR drug induced liver injury(MeSH] OR drug induced liver disease(MeSH] OR liver abnormalities, drug induced (MeSH] OR DILI(tiab] OR “toxic hepatitis”(tiab])	
#2 Search (new(tiab] OR novel(tiab])	
#1 Search (treatment*(tiab] OR therap*(MeSH] OR medicine*(MeSH] OR drug*(tiab] OR agent*(tiab])	

**FIGURE 1:**
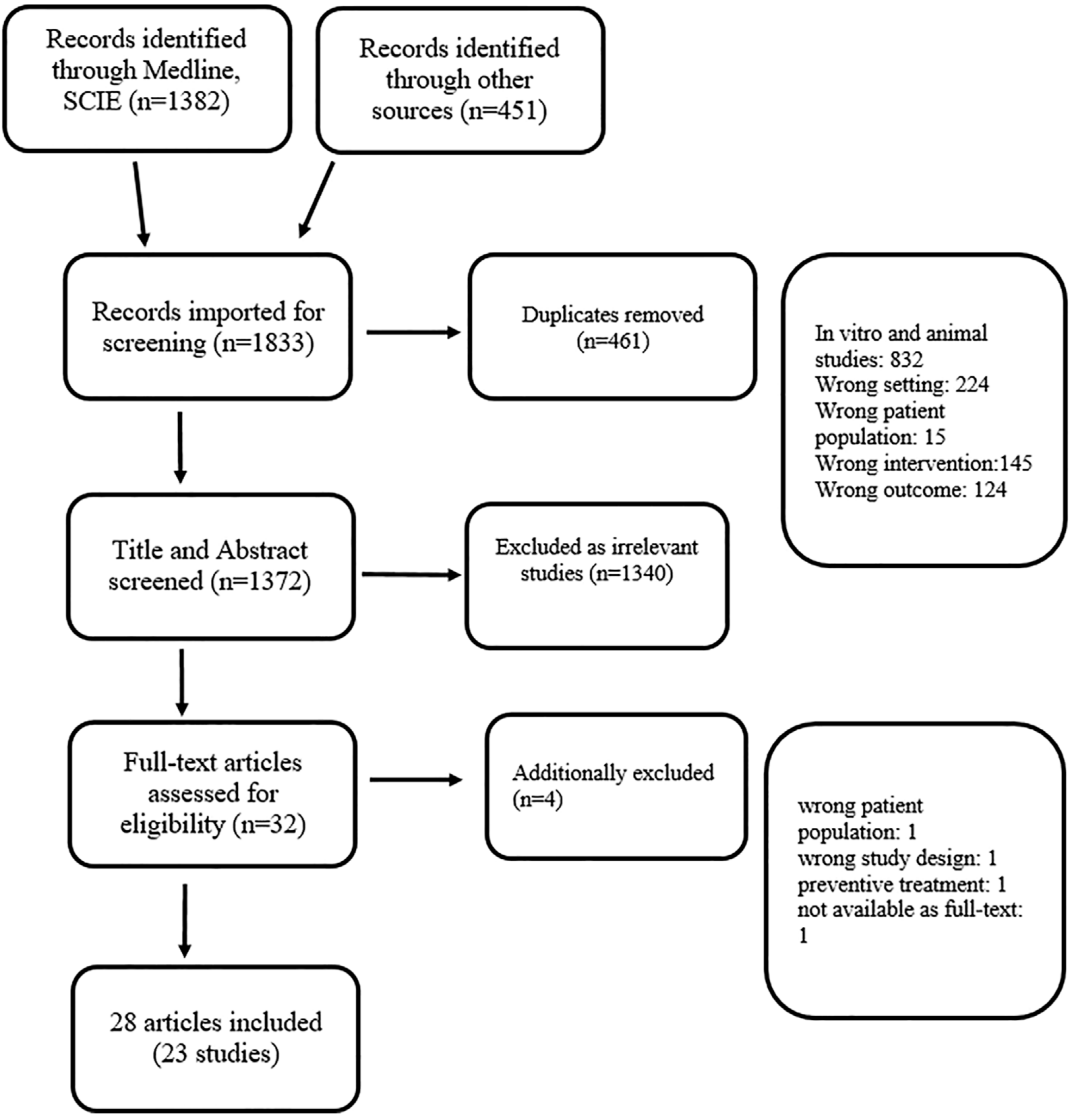
PRISMA flow diagram for systematic review, Novel therapies for the treatment of Drug-induced liver injury.

All articles (titles and abstracts) retrieved by the literature search were independently screened for eligibility by the review author based on the criteria described above. Full-text reports were obtained when studies appear to satisfy the inclusion criteria based on the title and abstract screening, or when information was insufficient to make a decision. Potential disagreements were resolved by consensus, or by referring to other review authors. The number of studies identified, excluded and included, are reported according to the PRISMA (the Preferred Reporting Items for Systematic Reviews and Meta-Analyses) checklist. The authors of the current review searched references of relevant articles retrieved by electronic searches, clinical trial databases, websites, and cross-referencing. In the case when information in an available paper was insufficient/incomplete, an attempt was made to get relevant data from the primary authors. A systematic literature review process is presented by the PRISMA flow diagram (Figure 1) (Moher et al., 2009).

The risk of bias in included studies was described in line with The Cochrane risk of bias tool (Higgins et al., 2021). Five domains (selection, performance and detection, attrition, reporting and other) were assessed and judged as introducing high, unclear or low risk of bias, based on supports for judgments from included studies.

The Grading of Recommendations, Assessment, Development and Evaluation (GRADE) criteria was employed to assess the certainty of the evidence for the primary outcomes (laboratory and/or clinically parameters of liver function) prespecified in the Method section or protocols of included studies. The GRADE approach involves five domains that were assessed: 1) risk of bias, 2) indirectness of evidence, 3) inconsistency, 4) imprecision of effect estimates and 5) publication bias. It could have resulted in one of four possible levels of certainty for a body of evidence for a given outcome: high (further research is very unlikely to change our confidence in the estimate of effect), moderate (further research is likely to have an important impact on our confidence in the estimate of effect and may change the estimate), low (further research is very likely to have an important impact on our confidence in the estimate of effect and is likely to change the estimate), and very low (any estimate of effect is very uncertain) (Higgins et al., 2021).

## Results

**FIGURE 2:**
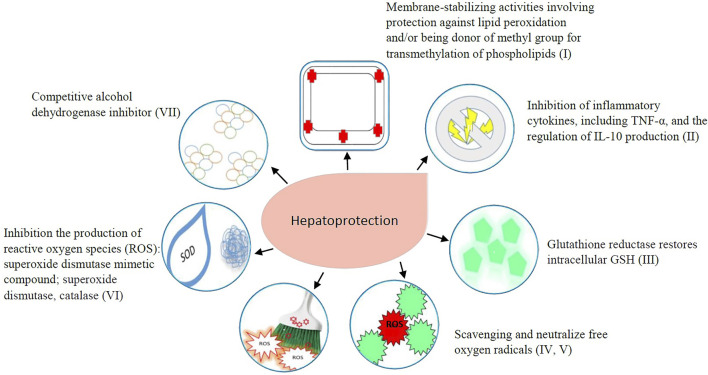
Mechanism of hepatoprotective effect of evaluated intervetions: Bicyclol: I, II, IV, V; Calmangafodipir: III, VI; Cytisin amidophosphate: I, IV, V; Livina: III, IV, V, VI; Magnesium Isoglycyrrhizinate: II, IV, V; Picroliv: I, IV, V, VI; Plasma Exchange: II; Radix Paeoniae Rubra: II, IV, V; S-adenosylmethionine: I, II, III; 4-Methylpyrazole: VII.

One thousand three hundred eighty-two articles were identified through the Medline (PubMed) and Science Citation Index Expanded databases. Additional 451 articles were identified through clinical trial registries, a website search, and cross-referencing. After duplicates were removed (*n* = 461), 1,372 titles and abstracts were screened of which 1,340 were excluded as irrelevant for this review. Thirty-two articles were assessed for eligibility as full-text of which four articles were excluded (Figure 1). Finally, a total of 28 publications were included in the current study. Included articles were described in Table 2 with the following characteristics: intervention, type of the study, country, participants, benefit in humans, adverse drug reactions, reference, offending drug(s), criteria for DILI or drug-induced toxic hepatitis diagnosis. Additionally, the mechanism of antioxidant, immunomodulation and other potentially hepatoprotective effect was described in Figure 2 for each included intervention.

**TABLE 2:** Characteristics of included studies.

Intervention	Type of the study	Country	Participants	Benefit in humans	Adverse drug reactions	References	The offending drug(s)	Criteria for DILI/drug induced toxic hepatitis diagnosis
Bicyclol	Randomized, double blind, positive controlled, multicenter phase II study	China	244	Results not published; Primary outcome measure: ALT decline range	Not applicable (NA)	NCT02944552 (2016)	NA	Acute DILI clinical diagnosis, patients with ALT 3–-20× ULN, TBL ≤2× ULN, liver biochemical abnormalities duration ˂90 days were included in the study
Bicyclol	Randomized, positive controlled, multicenter study	China	168	The ALT levels in the bicyclol group significantly lower vs. control group	No SAEs, no statistically significant differences between the two groups in AEs	Naiqiong et al. (2017)	Statins	Acute DILI clinical diagnosis, patients with ALT ≥ 2–5× ULN, TBL ≤2× ULN, liver biochemical abnormalities duration ˂3 months were included in the study
Calmangafodipir	Randomized open-label, rising-dose phase I study	United Kingdom	24	Good safety profile and reduced biomarkers of paracetamol toxicity (ALT, INR)	No AEs or SAEs were probably or definitely calmangafodipir-related	Morrison et al. (2019); The POP Trial Investigators and Dear (2019)	Paracetamol	No official definition, but hepatotoxicity was assessed using ALT and INR.
Cytisin amidophosphate	Randomized, double blind, positive controlled, single-center study	Kazakhstan	142	Normalization of hepatic inflammatory markers, increase activity of antioxidant enzymes	Tachycardia and hypertension related to high doses of cytisin amidophosphate	Zhumadilov et al. (2012)	Ethyl alcohol, alcohol surrogates, reserpine, paracetamol	No official definition, but patients with diagnosis of acute toxic hepatitis based on history, ultrasound and biochemical tests (ALT, AST, TBL) were included in the study
Fomepizole	Randomized, double-blind, cross-over, single centre, study	United States	5	Reduced oxidative metabolism and NAPQI production after paracetamol overdose	NA	Kang et al. (2020)	Paracetamol	DILI was not considered, but paracetamol and its metabolites
Fomepizole	Case report, case series	United States	8	ALT, AST and INR normalisation and/or significant decrease of paracetamol plasma concentration	Not reported	Zell-Kanter et al. (2013); Rampon et al. (2020); Shah and Beuhler (2020)	paracetamol, ethanol, benzodiazepine, diphenhydramine, salicylate, ibuprofen, loperamide	No official definition, but patients with increased AST and ALT levels and/or elevated paracetamol plasma concentration
Livina^a^	Randomized, single blind, placebo controlled study	India	42	AST, ALT significantly lower in the Livina vs. placebo group	None related to the Livina treatment	Gulati et al. (2010)	Antituberculotic drugs: rifampicin, Isoniazid, ethambutol, pyrazinamide	No official definition, but efficacy of the Livina or placebo was assessed by performing liver function tests (serum bilirubin, AST, ALT and ALP)
Magnesium Isoglycyrrhizinate (MgIG)	Randomized, double-blind, active controlled, multidose, multicentre phase II study	China	174	ALT normalization in MgIG groups (either dose) significantly greater vs. active control group	No significant difference in safety	Wang et al. (2019)	Antituberculotic drugs, antitumor, traditional Chinese medicine, antibiotics, cardiovascular,anti-inflammatory, hormone	No official definition, but patients with ALT ≥ 2× ULN, TBL ≤ 3× ULN, liver biochemical abnormalities duration ≤3 months were included in the study
MgIG	Randomized, single-blind, controlled, multicentre phase IV study	China	73	Results not published; Primary Outcome Measure: ALT normalization rate	NA	NCT04595916 (2020)	NA	Acute DILI clinical diagnosis, patients with ALT ≥ 3× ULN, TBL ≤5× ULN, duration of current liver injury ˂6 months were included in the study
S-adenosylmethionine	Retrospective study	Italy	233	Reduction of AST, ALT, LDH, and TBL, AP, GGT, liver toxicity grade, minimal number of chemotherapy dose reductions or administration delays	Not reported	Vincenzi et al. (2011); Vincenzi et al. (2012)	Chemotherapy regimens^b^ (FOLFIRI, CMF, FOLFOX, Bevacizumab+XELOX)	No official definition, but patients with AST or/and ALT ≥ 2.5× ULN, were included in the study
	Prospective study					Santini et al. (2003)		Liver toxicity was assessed according to NCI-CTCAE^c^ (version 3), course delays, discontinuations and dose reductions due to liver toxicity were recorded
Picroliv	Randomized, placebo controlled study	India	260 in protocol, but 182 in abstract	Protocol is available online, results in abstract form only—inconsistent	NA	Gaur and Bhosale, (2002); Bhosale et al. (2013)	Antituberculotic drugs	No official definition
Plasma exchange (TPE)	Retrospective study	India	10	Significant improvement in aminotransferases, TBL, direct bilirubin, INR	None related to the TPE.	Sachan et al. (2017); Jothimani et al. (2018)	Antituberculotic drugs, antimalarial drugs with paracetamol overdose, native medication for skin and rheumatic disorders, stanazol, carbamazepine, Augmentin	No official definition, but patients with bilirubin >15 mg/dl and diagnosed as DILI with history of recent drug intake were included in study
TPE	Case reports, case series, Letter to the editor	Turkey, Germany, India, Spain, China	19	Pathological, laboratory and clinical markers of liver function have been improved	None reported and related to TPE	Aydemir et al. (2005); Bilgir et al. (2013); Liu et al. (2013); Göpel et al. (2016); Philips et al. (2017); Riveiro-Barciela et al. (2019); Rong et al. (2020)	Propylthiouracil, L-asapaginase, PEG-asparaginase, metoprolol, ipilimumab, complementarymedication containing Fructus Psoraleae	Liver scintigraphy, liver function tests, liver biopsy, NA
TPE	Randomized controlled trial	India	30	Significant reduction in bilirubin, bile acid levels, INR and IL-6/TNF-α/IL-1β	Hypocalcaemia (59.2%) and alkalosis (42.9%) were the major adverse events; one patient had TRALI during TPE	Sinha et al. (2020)	Complementary and alternative medicines, not known for all participants	Diagnosis of severe DILI, based on history and liver biopsy and graded for the severity as by DILIN
Radix Paeoniae Rubra (RPR)	Prospective study	China	14	The levels of ALT, AST, TBL, direct bilirubin, total bile acid, Child-Pugh and MELD^d^ scores significantly decreased; the levels of albumin and cholinesterase statistically significantly elevated. Improved jaundice, fatigue	Diarrhea in six cases, but spontaneously disappeared after the RPR discontinuation	Jing et al. (2017)	Different spectra of drugs, including antibiotics, paracetamol, alternative medications	No DILI definition, no data about causality assessment

a Polyherbal preparation of 50 mg each of *Picrorhizha kurroa* (kutaki), *Phyllanthus niruri* (bhuyamalaki), *Andrographis paniculata* (kalmegh), *Cichorium invitybus* (kasni), *Tephrosia purpurea* (sharphaunka), *Solanum dulcamara* (kakamarchi), *Crenum aciaticum* (macchaka), *Astonia seholanis* (saptaparna), and 25 mg each of *Holarrhave ntidysentric* (indriyava), *Tinospora cordifolia* (guduchi), *Terminala chebula* (Haritaki), *Asteracantha longifolia* (kakilakshya).

b FOLFIRI regimen, Raltitrexed, Oxaliplatin, Irinotecan, 5-Fluorouracil (5-FU), Folinic Acid (FA); CMF regimen, Cyclophosphamide (CTX), Methotrexate (MTX) plus 5-FU; FOLFOX regimen: Oxaliplatin, FA, 5-FU; Bevacizumab + XELOX regimen: Bevacizumab, Oxaliplatin, Capecitabine.

c NCI-CTCAE: National Cancer Institute—The NCI Common Terminology Criteria for Adverse Events.

d MELD: model for end-stage liver disease.

### Description of Included Studies

We aimed to analyze data of 26 studies that were based on 28 publications, including full-text articles, abstracts only, protocols and data from clinical trial database. The full-text articles were published for six RCTs (Gulati et al., 2010; Zhumadilov et al., 2012; Naiqiong et al., 2017; Morrison et al., 2019; Wang et al., 2019; Kang et al., 2020), for two observational retrospective studies (Vincenzi et al., 2011; Vincenzi et al., 2012), for six case reports (Aydemir et al., 2005; Bilgir et al., 2013; Liu et al., 2013; Zell-Kanter et al., 2013; Philips et al., 2017; Shah et al., 2020), for two case series (Rampon et al., 2020; Rong et al., 2020), and for two letters to the editors (Gopel et al., 2016; Riveiro-Barciela et al., 2019). In abstract form only we found six studies: two RCTs (Bhosale et al., 2013; Sinha et al., 2020), two prospective observational studies (Santini et al., 2003; Jing et al., 2017) and one retrospective observational study (Sachan et al., 2017; Jothimani et al., 2018). The abstracts by Sachan et al. (2017) and Jothimani et al. (2018) reported the same study data. The only difference that we noted was Jothimani et al. (2018) defined study as retrospective and Sachan et al. (2017) as prospective. Protocols were published for two RCTs (Gaur and Bhosale, 2002; The POP Trial Investigators and Dear, 2019). The POP Trial Investigators and Dear (2019) protocol was related to the full-text article by Morrison et al. (2019). The protocol published by Gaur and Bhosale (2002) was related to the abstract by Bhosale et al. (2013). Data for two RCTs based on clinical trial database did not include results (NCT 02944552, 2016; NCT04595916, 2020) and we were not able to assess the risk of bias and certainty of evidence for those studies.

Most studies defined DILI on a range of liver enzymes alanine aminotransferase (ALT), aspartate aminotransferase (AST), alkaline phosphatase (ALP) or total bilirubin (TBL) elevations from approximately 1.25 to 5-fold upper limit of normality (ULN), and three studies with S-adenosylmethionine (SAMe) used the definition in the National Cancer Institute - The NCI Common Terminology Criteria for Adverse Events (NCI-CTCAE) version 3.0. The use of liver-specific causality assessment scales was limited to four RCTs involving Roussel Uclaf Causality Assessment Method (RUCAM) (bicyclol, magnesium isoglycyrrhizinate (MgIG)). Main efficacy criteria in DILI management was the decrease or normalization of the liver laboratory parameters, or survival rate in DILI-related ALF patients. Main characteristics and methodologic quality assessment of each of publication analyzed are summarized in Tables 2–4.

**TABLE 3:** Risk of bias across included studies.

Study	Selection bias (random sequence generation and allocation concealment)	Performance and detection bias (blinding to participants, personnel and outcome assessment)	Attrition bias (incomplete outcome data)	Reporting bias (selective reporting)	Other bias (including sample size estimation and intention-to-treat analysis)
Bicyclol					
Naiqiong et al. (2017)	L	U	L	L	U
Calmangafodipir					
Morrison et al. (2019)	L	H	L	L	H
Cytisin amidophosphate					
Zhumadilov et al. (2012)	U	U	L	L	U
Fomepizole					
Kang et al. (2020)	L/U	U	L	L	H
Zell Kanter et al. (2013)	H	H	U	U	U
Shah and Beuhler (2020)	H	H	U	U	U
Rampon et al. (2020)	H	H	U	U	U
Livina					
Gulati et al. (2010)	L	H	U	L	U
Magnesium Isoglycyrrhizinate					
Wang et al. (2019)	U	U	L	L	H
Picroliv					
Bhosale et al. (2013)	U	U	U	H	U
Plasma exchange					
Jothimani et al. (2018)	H	H	U	H	U
Aydemir et al. (2005)	H	H	U	H	U
Bilgir et al. (2013)	H	H	U	H	U
Liu et al. (2013)	H	H	U	H	U
Göpel et al. (2016)	H	H	U	H	U
Philips et al. (2017)	H	H	U	H	U
Riveiro-Barciela et al. (2019)	H	H	U	H	U
Rong et al. (2020)	H	H	U	H	U
Sinha et al. (2020)	H	H	L	U	U
Radix Paeoniae Rubra					
Jing et al. (2017)	H	H	U	U	U
S-adenosylmethionine					
Vincenzi et al. (2011)	H	H	U	L	L
Vincenzi et al. (2012)	H	H	U	L	U
Santini et al. (2003)	H	H	U	L	U

**TABLE 4:** Summary of main outcomes and the certainty of the evidence.

Primary outcome/follow-up time	Results		No of participants/studies	Certainty of the evidence (GRADE)	Comments
	Active treatment	Control treatment			
ALT (mean ± SD; U/L)/28 days	Bicyclol 30.36 ± 17.41	Polyene phosphatidylcholine 50.71 ± 27.13	168/1 RCT	Low^a^	A statistically significant difference was assessed at *p* <0.01
Proportion of patients experienced (S)AEs (%)/90 days	Calmangafodipir + NAC	NAC	24/phase 1 study	Very low^b^	Active treatment involved three patient’s group: 2, 5 or 10 μmol/kg
	AEs: 100% (18/18)	AEs: 6/6 (100%)			
	≥1 SAES: 50% (9/18)	≥1 SAES: 33.33% (2/6)			
ALT (nmol/L); AST (nmol/L); bilirubin (mcmol/l) average ± SD/3 days	Cytaphat	Essentiale	142/1 clinical study	Very low^c^	Control treatment involved two patient’s groups. The statistically significant difference was observed between Cytaphat and placebo group (*p* <0.05)
	ALT 248.9 ± 35.8	ALT 232.2 ± 20.8			
	AST 109.1 ± 8.7	AST 154.7 ± 19.6			
	Bilirubin 19.8 ± 1.7	Bilirubin 24.6 ± 2.4			
		Placebo			
		ALT 354.7 ± 45			
		AST 297.1 ± 33.6			
		Bilirubin 34.5 ± 3.7			
Fraction of ingested paracetamol excreted as total oxidative metabolites/24-h urine	Fomepizole + paracetamol 80 mg/kg	paracetamol 80 mg/kg	6/1 clinical crossover study	Very low^d^	All participants were healthy volunteers. Mean difference between groups was 3.97%, 95% CI 2.31–5.63%, *p* = 0.003)
	0.51%	4.48%			
ALT (U/L); AST (U/L); Bilirubin, INR	Fomepizole + NAC	No control	8/3 case studies	Very low^e^	All participants were paracetamol overdosed. Data were based on one case series (6 cases) and 2 case reports
ALT (IU/L); AST (IU/L); ALP (U/L); Bilirubin (mg/dl)/8 weeks	Livina	Placebo	42/1 study	Very low^f^	All participants were treated for TBC. The statistically significant difference was observed for AST, ALT, ALP in placebo group compared to itself baseline (*p* <0.05)
	ALT 28.7 ± 8.4	ALT 52.5 ± 7.6			
	AST 30.1 ± 8.3	AST 51.9 ± 10.5			
	ALP 239.1 ± 19.7	ALP 386.2 ± 29.3			
	Bilirubin 0.95 ± 0.3	Bilirubin 1.46 ± 0.5			
Proportion of ALT normalization/28 days	MgIG 100 mg 85.71% (50/59 subjects)	Tiopronin 61.02% (36 subjects)	174/1 RCT	Moderate^g^	The difference between MgIG 100 mg and tiopronin and between MgIG 200 mg and tiopronin were significant (*p* = 0.0111; *p* = 0.0087). Although, the results stayed significant, calculated proportions for active treatment in the originally publication were mixed: 85.71% corresponds to 48 out of 56 participants
	MgIG 200 mg 84.75% (48/56 subjects)				
The number of patients who develop hepatotoxicity	Picroliv	Placebo	182/1 study in abstract form only	Very low^h^	Results published in abstract significantly differ from those prespecified in protocol: there was no reported hepatotoxicity related to picroliv treatment
	NA	NA			
Not prespecified: AST, ALT, bilirubin, INR.	PE	No control	10/1 study in abstract form only	Very low^i^	The level of AST, ALT, bilirubin and INR have not been shown
	There was significant improvement in aminotransferases (*p* < 0.05), direct bilirubin (*p* < 0.001), INR (*p* < 0.05)				
<20% reduction in serum bilirubin from baseline after 3 TPE sessions or reduction in serum bilirubin by < 5 mg and INR <1.5/28 days	PE	SMT	30/1 study in abstract form only	Very low^j^	No measurement unit specified for bilirubin level; SMT not defined. Significant difference in bilirubin when compared to baseline levels, *p* <0.002
	bilirubin	Bilirubin			
	5.3 ± 7.6	10.4 ± 8.9			
Different laboratory parameters and clinical symptoms of liver function	PE	No control	19/4 case reports, one case series and two letter to the editors	Very low^k^	
Primary outcome not prespecified. AST (U/L), ALT, TBL (mg/dl), direct bilirubin (mg/dl), total bile acid (µmol/L), Child-Pugh and MELD scores; different clinical symptoms/3–6 months after RPR treatment	RPR	No control	14/1 study abstract form only	Very low^l^	All laboratory parameters were statistically significantly decreased, *p* <0.05. The outcomes were compared before and after RPR treatment in the same patient
	AST 113 ± 77 vs 49 ± 29				
	ALT 101 ± 91 vs. 38 ± 35				
	TBL 20.6 ± 6.1 vs. 4.9 ± 8.2				
	direct bilirubin 15.2 ± 5.3 vs. 3.2 ± 6.1				
	total bile acid 282 ± 134 vs. 50 ± 74				
	Child-Pugh 8.5 ± 1.1 vs. 6.3 ± 2.0 MELD score 25.0 ± 2.5 vs. 15.6 ± 6.6				
	jaundice (100 vs. 21%; *p* <0.001); fatigue (86 vs. 29%; *p* =0.006)				
AST, ALT, LDH, GGT, ALP, TBL/follow-up time not prespecified	SAMe	Placebo	233/3 study	Very low^m^	Data from one study was available only in abstract as corresponding author did not reply to our email
					The patient group (183) consisted of patients with colorectal cancer and treated with FOLFOX or B-XELOX chemotherapy regiments. Additional 50 patients had unspecified cancer and chemotherapy regimen was not described in abstract
					All prespecifed outcomes were found to be statistically significantly reduced in the SAMe group, *p* at least ≤0.04)

ALP, alkaline phosphatase; 4-MP, fomepizole or 4-Methylpyrazole; GGT, gammaglutamyltransferase; INR, international ratio; LDH, lactate dehydrogenase; MELD, model for end-stage liver disease; MgIG, magnesium isoglycyrrhizinate; NA, not applicable; NAC, N-acetylcistein; paracetamol (acetaminophen or N-Acetyl-p-Aminophenol); PE, plasma exchange; RCT, randomized control trial; RPR, Radix Paeoniae Rubra; (S)AEs, (serious) adverse events; SAMe, S-adenosylmethionine; SD, standard deviation; SMT, standard medical treatment; TBL, total bilirubin; U/L, unit per litre.

a Downgraded twice for serious indirectness and imprecision (not representative sample: all participants had statin-induced liver injury and results are based on single study data included less than 400 patients.).

b Downgraded three times for high risk of bias; not generalizable results (indirectness) and very sparse data (small sample size).

c Downgraded three times for serious risk of bias, indirectness of the evidence (aetiology of acute toxic hepatitis included and only demonstrated statistically significant difference at the level *p* < 0.05 between Cytaphat and placebo) and very sparse data (small sample size).

d Downgraded four times for serious risk of bias, serious indirectness of the evidence (only healthy volunteers included) and very serious imprecision (very sparse data).

e Downgraded six times for very serious risk of bias (data based on case reports studies), serious inconsistency (heterogeneity between reports was remarkable in baseline characteristics, treatment administered and all work-up done), indirectness of the evidence (paracetamol overdosed all) and very serious imprecision (very sparse data).

f Downgraded four times for serious risk of bias, indirectness of the evidence (TBC patients only included) and very serious imprecision (very sparse data).

g Downgraded once for serious imprecision due to sparse data (small sample size, patients divided into three subgroup).

h Downgraded four times for very serious risk of bias (outcomes were not reported as prespecified), indirectness of the evidence (TBC patients only) and publication bias (only abstract form published).

i Downgraded six times for very serious risk of bias, serious inconsistency (heterogeneity could not be assessed due to lack of data), very serious imprecision (very sparse data) and publication bias.

j Downgraded seven times for very serious risk of bias, very serious inconsistency (heterogeneity could not be assessed due to lack of data; prespecified outcome described in the Methods was not described in Results), and very serious imprecision (very sparse data) and publication bias (only abstract form published).

k Downgraded six times for very serious risk of bias (case reports and series), very serious inconsistency (considerable heterogeneity within the patient group, treatment and follow-up), and very serious imprecision (very sparse data).

l Downgraded seven times for very serious risk of bias, serious inconsistency and indirectness (heterogeneity and generalizability could not be assessed due to lack of data), very serious imprecision (very sparse data) and publication bias (only abstract form published).

m Downgraded four times for very serious risk of bias, serious indirectness (only cancer patients included), and publication bias (only abstract available for one study).

### Participants

A total number of 1,051 participants, including healthy volunteers and patients receiving offending drug, were evaluated. Baseline demographic, laboratory and clinical characteristics were defined by inclusion and exclusion criteria of included studies and stratified similarly between active and control groups in RCTs. The difference in participants’ baseline characteristics was more notable in observational descriptive studies.

The etiology of DILI was related to paracetamol overdose, antituberculotics, statins, alcohol, reserpine, traditional Chinese medicines, different antibiotics, cardiovascular drugs, anti-inflammatory drugs, hormones, different chemotherapeutic protocols (bevacizumab plus oxaliplatin, capecitabine, or oxaliplatin, folinic acid, 5-fluorouracil), and paracetamol concomitantly ingested with benzodiazepine, diphenhydramine, salicylate, ibuprofen, or loperamide (Tables 2, 4).

### Outcomes

Outcomes measures have not been homogenous within included studies, and mainly refer to changes in laboratory findings of liver function. Additionally, a clinical evaluation of liver function was reported in included studies, but marked heterogeneity was detected with the emphasis that pathological presentation, positive signs and symptoms, were more frequently described. With respect to the methodological quality of observational studies, there was no standard form of reporting for laboratory markers and clinical presentation of included patients. The incidence of (serious) adverse events ((S)AEs) as a primary outcome was described in only one study evaluating calmangafodipir (Table 4).

### Risk of Bias in Included Studies

Risk of bias was described in detail in Table 3 and in Supplementary Table S1. Here we present summarized risks for every defined domain for every included study. Studies designed as case reports, case series and letters to the editor for the same intervention were grouped and assessed as single study (first group of studies: Zell-Kanter et al., 2013, Shah and Beuhler, 2020; Rampon et al., 2020; second group of studies: Santini et al., 2003, Vincenzi et al., 2011, Vincenzi et al., 2012; third group of studies: Aydemir et al., 2005; Bilgir et al., 2013; Liu et al., 2013; Göpel et al., 2016; Philips et al., 2017; Riveiro-Barciela et al., 2019; Rong et al., 2020).

#### Selection Bias (Random Sequence Generation and Allocation Concealment)

Three studies were assessed as introducing low risk of bias for this domain (Gulati et al., 2010; Naiqiong et al., 2017; Morrison et al., 2019). In four studies it was unclear if selection bias was introduced (Zhumadilov et al., 2012; Bhosale et al., 2013; Wang et al., 2019; Kang et al., 2020). We judged that six studies introduced high risk of bias (first group of studies: Zell-Kanter et al., 2013, Shah and Beuhler, 2020, Rampon et al., 2020; second group of studies: Santini et al., 2003; Vincenzi et al., 2011; Vincenzi et al., 2012; third group of studies: Aydemir et al., 2005; Bilgir et al., 2013; Liu et al., 2013; Göpel et al., 2016; Jing et al., 2017; Philips et al., 2017; Jothimani et al., 2018; Riveiro-Barciela et al., 2019; Rong et al., 2020; Sinha et al., 2020).

#### Performance and Detection Bias (Blinding of Participants, Personnel and Outcome Assessment)

No study introduced low risk of bias for this domain. Unclear risk of bias we assessed in five studies: Zhumadilov et al. (2012), Bhosale et al. (2013), Naiqiong et al. (2017), Wang et al. (2019), and Kang et al. (2020). Eight studies were judged as introducing high risk of bias: Morrison et al. (2019); first group of studies: Zell-Kanter et al. (2013), Rampon et al. (2020), and Shah and Beuhler (2020) second group of studies: Santini et al. (2003), Vincenzi et al. (2011), and Vincenzi et al. (2012); third group of studies: Aydemir et al. (2005), Gulati et al. (2010), Bilgir et al. (2013), Liu et al. (2013), Göpel et al. (2016), Jing et al. (2017), Philips et al. (2017), Jothimani et al. (2018), Riveiro-Barciela et al. (2019), Rong et al. (2020), and Sinha et al. (2020).

#### Attrition Bias (Incomplete Outcome Data)

Six studies were judged as free of bias for this domain (Zhumadilov et al., 2012; Naiqiong et al., 2017; Morrison et al., 2019; Wang et al., 2019; Kang et al., 2020; Sinha et al., 2020). It was unclear for seven studies (first group of studies: Zell-Kanter et al., 2013, Shah and Beuhler, 2020; Rampon et al., 2020; second group of studies: Santini et al., 2003, Vincenzi et al., 2011, Vincenzi et al., 2012; third group of studies: Aydemir et al., 2005; Gulati et al., 2010; Bhosale et al., 2013; Bilgir et al., 2013; Liu et al., 2013; Göpel et al., 2016; Jing et al., 2017; Philips et al., 2017; Jothimani et al., 2018; Riveiro-Barciela et al., 2019; Rong et al., 2020) if there were attrition in outcome data. No study was judged as introducing high bias considering this domain.

#### Reporting Bias (Selective Reporting)

A low risk of bias for selective reporting domain was judged in seven studies (Zhumadilov et al., 2012; Naiqiong et al., 2017; Morrison et al., 2019; Wang et al., 2019; Kang et al., 2020; Gulati et al., 2010; second group of studies: Santini et al., 2003; Vincenzi et al., 2011; Vincenzi et al., 2012). It was unclear if there was a risk of bias in three studies (first group of studies: Zell-Kanter et al., 2013; Jing et al., 2017; Rampon et al., 2020; Shah and Beuhler, 2020; Sinha et al., 2020). Three studies were judged as introducing high risk of bias (Bhosale et al., 2013; Jothimani et al., 2018; third group of studies: Aydemir et al., 2005; Bilgir et al., 2013; Liu et al., 2013; Göpel et al., 2016; Philips et al., 2017; Riveiro-Barciela et al., 2019; Rong et al., 2020).

#### Other Bias

No study was assessed as introducing low risk of bias. In 10 studies it was unclear if other biases were present (Zhumadilov et al., 2012; Naiqiong et al., 2017; first group of studies: Gulati et al., 2010; Zell-Kanter et al., 2013; Shah and Beuhler, 2020; Rampon et al., 2020; second group of studies: Santini et al., 2003; Vincenzi et al., 2011; Vincenzi et al., 2012; third group of studies: Aydemir et al., 2005; Bhosale et al., 2013; Bilgir et al., 2013; Liu et al., 2013; Göpel et al., 2016; Jing et al., 2017; Philips et al., 2017; Jothimani et al., 2018; Riveiro-Barciela et al., 2019; Rong et al., 2020; Sinha et al., 2020). A high risk of bias was judged in three studies: Morrison et al. (2019), Wang et al. (2019), and Kang et al. (2020).

### Effect of Interventions

Bicyclol, calmangafodipir, cytisin amidophosphate, fomepizole, Livina, MgIG, picroliv, plasma exchange treatment, radix Paeoniae rubra (RPR) and S-adenosylmethionine (SAMe) were investigated as the active, intervention arm compared to control arm mostly including either the standard medical treatment (SMT), polyene phosphatidylcholine, NAC, tiopronin, or placebo (Table 2).

Two interventions investigated in RCTs were authorized in China. Bicyclol is authorized for the treatment of elevated aminotransferase caused by chronic hepatitis (Niu et al., 2021). Magnesium isoglycyrrhizinate is authorized by the China Food and Drug Administration for the treatment of acute DILI and chronic viral hepatitis (Niu et al., 2021). None of included interventions was approved by European Medicine Agency in the European Union.

We assessed quality of the evidence (certainty) for primary outcomes of 10 therapeutic interventions based on included studies. Studies designed as case reports, case series and letters to the editor for the same intervention were grouped and assessed as single study (first group of studies: Zell-Kanter et al., 2013, Shah and Beuhler, 2020, Rampon et al., 2020; second group of studies: Santini et al., 2003, Vincenzi et al., 2011, Vincenzi et al., 2012 and third group of studies: Aydemir et al., 2005; Bilgir et al., 2013; Liu et al., 2013; Göpel et al., 2016; Philips et al., 2017; Riveiro-Barciela et al., 2019; Rong et al., 2020). In studies published in abstract form only and case studies primary outcomes were not prespecified and we noticed that in Table 4. Additional detailed information is shown in Table 4.

#### Bicyclol

Bicyclol (4,4′-dimethoxy-5,6,5′,6′-bis (methylenedioxy)-2-hydroxymethyl-2′-methoxycarbonyl biphenyl) is a synthetic drug used as a protectant against various liver diseases in China and in other countries (Liu, 2009). Its mechanism of action is related to a decrease in free radical-induced damage to hepatocytes; scavenging free radicals, protection against lipid peroxidation, protection of hepatic cell membranes and mitochondrial function and inhibition of inflammatory cytokines (Figure 2) (Liu et al., 2005; Wang and Li, 2006). Previous research has shown that bicyclol demonstrated a marked hepatoprotective effect, reduced elevated serum transaminase levels, reversed drug-induced pathological changes, and promoted tissue repair (Liu et al., 2005; Wang and Li, 2006; Xie et al., 2012; Han et al., 2014). The family of CYP3A/2E1 is involved in its biotransformation, thus no evident pharmacokinetic interactions have been reported with different medications. Toxicity investigations did not demonstrate significant biochemical blood changes or histopathological alteration of various organs during the chronic treatment with bicyclol. All these aspects indicate that bicyclol may be a valuable agent for acute, chronic and degenerative liver pathology (Zhao et al., 2021).

We aimed to include two studies on bicyclol in this review. The first was a RCT in 168 patients with statin-induced liver injury and RUCAM score ≥6 investigating the efficacy and safety of bicyclol treatment (Naiqiong et al., 2017). Patients were equally randomized to the treatment group to receive bicyclol (25 mg; *N* = 79) or to the control group to receive a polyene phosphatidylcholine, a drug for the auxiliary improvement of toxic liver damage approved in China (456 mg; *N* = 78). Both substances were given orally, three times a day (TID) for 4 weeks. The primary endpoint was a decrease of baseline ALT serum levels, and secondary endpoints included serum ALT normalization rates after two and 4 weeks of treatment and safety assessment.

The second study was a phase II RCT to explore the efficacy and safety of different doses of bicyclol tablets (25 mg or 50 mg TID) in treatment of acute DILI compared to polyene phosphatidylcholine capsule (456 mg TID) in patients with RUCAM score ≥6. This study was completed in 244 patients in China in 2020, but currently, there are no results available (NCT02944552, 2016).

The effect of bicyclol was compared to polyene phosphatidylcholine in one study. A low certainty evidence suggests bicyclol results in a reduction of ALT level (30.36 ± 17.41 vs 50.71 ± 27.13 U/L) after 4 weeks of treatment, *p* < 0.01.

#### Calmangafodipir

A specific calcium-manganese mixed metal complex of N, N′-bis-(pyridoxal-5-phosphate)-ethylenediamine-N, N′-diacetic acid is calmangafodipir, also known as Ca_4_MnDPDP_5._ It is a superoxide dismutase mimetic compound, derivative of the magnetic resonance imaging contrast agent mangafodipir and containing approximate calcium to manganese molar ratio of 4:1 (Karlsson et al., 2015). It mediates the protection of liver cells from oxidative stress generated by high doses of paracetamol, especially in the late stages (Jaeschke et al., 2020; Mullins et al., 2020; Rosa et al., 2021).

A single phase I study that we included in review was a randomized, open-label, exploratory study evaluating the safety, tolerability and efficiency of calmangafodipir co-treatment with NAC compared with the NAC alone classic regimen in 24 patients with paracetamol overdose. Patients were randomized into four subgroups receiving single intravenous dose of calmangafodipir 2, 5, or 10 μmol/kg plus NAC (N = 6 patients in each group) versus placebo group (*N* = 6). The protocol of this trial is available online and all prespecified endpoints were investigated in the trial (The POP Trial Investigators and Dear, 2019). No data about DILI causality assessment was available. Primary outcomes were safety profile, and tolerability of calmangafodipir. Secondary outcomes included the level of ALT, international normalized ratio (INR), keratin-18, caspase-cleaved keratin-18 (ccK18), microRNA-122, and glutamate dehydrogenase (Morrison et al., 2019).

No significant difference was found in proportion of patients experienced (S)AEs in 90 days’ follow-up period between group of patients overdosed with paracetamol and treated with calmangafodipir and NAC versus NAC alone (AEs 100% for both groups; ≥ 1 SAEs: 50 vs 33.33%), but the evidence is very uncertain about the effect of calmangafodipir.

#### Cytisin Amidophosphate

Cytisin amidophosphate (O, O Dimethyl-N-cytinizylphosphate) is a phosphor-derivative of alkaloid cytosine, synthesized in Kazakhstan and used under the name Cytaphat. It has antioxidant, membrane stabilizing and cholagogic properties, proved by preclinical studies. Cytoprotective properties were noted in acute toxic hepatitis, chronic hepatitis and jaundice (Zhumadilov et al., 2012). To assess the efficacy and safety of Cytaphat, Zhumadilov et al. carried out a phase II, double-blind RCT in 142 patients with acute toxic hepatitis, caused by different drugs (ethyl alcohol, alcohol surrogates, reserpine, paracetamol). No data about DILI causality assessment was available. Patients were randomized either to receive Cytaphat (10 mg/kg; *N* = 94), placebo (10 mg; *N* = 14) or standard hepatoprotector, purified phosphatidylcholine extract from soybean, under the name Essentiale (10 ml; *N* = 34). All substances were administered intravenously, once a day for 3 days. The outcomes included the level of ALT, AST, and bilirubin before and after the treatment; activity of antioxidant enzymes, and the average time of the elimination of toxic hepatopathy (days). (Zhumadilov et al., 2012).

Very low-certainty evidence suggests a significant decrease in ALT, AST, and bilirubin levels comparing each, cytisin amidophosphate group (248.9 ± 35.8 nmol/sl, 109.1 ± 8.7 nmol/sl, 19.8 ± 1.7 μM/L), and phosphatidylcholine group (232.22 ± 0.8 nmol/sl, 154.71 ± 9.6 nmol/sl, 18.62 ± 0.4 μM/L) to placebo group (354.7 ± 25.0 nmol/sl, 297.1 ± 33.6 nmol/sl, 34.5 ± 3.7 μM/L; *p* < 0.05).

#### Fomepizole

Fomepizole, 4-methylpyrazole or 4-MP, is a competitive alcohol dehydrogenase inhibitor used as an antidote for toxic ethylene glycol and methanol poisoning (approved by the Food and Drug Administration) and is known as a potent and specific CYP2E1 inhibitor.

The results of an animal study of paracetamol overdose-induced liver injury showed that fomepizole was effective in the amelioration of hepatotoxicity grade, and degrees of hepatic necrosis when administered in combination with NAC (Akakpo et al., 2018). Fomepizole was administered in single dose of 50 mg/kg (equivalent dose in humans is 4.1 mg/kg that is less than the clinically approved dose of 10–15 mg/kg every 12 h) after a toxic dose of paracetamol, and prevented mitochondrial dysfunction, hepatocytes necrosis, and protein adducts formation as well as glutathione (GSH) depletion.

Fomepizole is not only effective in preclinical animal experiments but also in human hepatocytes, in human volunteers, and patients with paracetamol-induced liver injury (Akakpo et al., 2020). Zell-Kanter et al. (2013) reported a case report of a female patient with paracetamol overdose who was efficiently treated with intravenous NAC and empirically with a single dose of 4-methylpyrazole administered due to suspicion of simultaneous alcohol poisoning. In human hepatocytes fomepizole inhibited CYP2E1, blocked paracetamol activation, and N-acetyl-p-benzoquinone imine (NAPQI) formation, and downstream pathways that lead to necrosis of hepatocytes (Akakpo et al., 2018). Based on these results, a clinical crossover study with healthy volunteers given 80 mg/kg paracetamol with or without fomepizole (15 mg/kg intravenously) was performed (Kang et al., 2020). The reduction in oxidative metabolites indicating effective inhibition of paracetamol reactive metabolite NAPQI production and safety of fomepizole treatment with a mild overdose of paracetamol were described. Fomepizole showed positive or even slightly better effect than NAC in preventing reactive metabolite formation and mitochondrial oxidant stress.

Following the first report, there was an increasing number of case reports published on the off-label use of fomepizole in the treatment of acute liver injury and prevention of liver failure in patients overdosed with paracetamol and concomitant ingestion of other drugs such as benzodiazepine, diphenhydramine, salicylate, ibuprofen, and loperamide (Rampon et al., 2020; Shah and Beuhler, 2020). All the patients received NAC within 8–9 h of ingestion, and the decision to administer fomepizole was based on the significant increase in AST/ALT and/or paracetamol plasma concentrations similar to those seen in patients who developed liver injury despite early NAC therapy. However, no criteria were used to confirm DILI. Single dose of fomepizole (15 mg/kg intravenously) led to biochemical and clinical recovery (Shah and Beuhler, 2020). Despite persistently elevated paracetamol plasma concentrations, co-administration of fomepizole (15 mg/kg intravenously followed by 10 mg/kg 12 h later), resulted in ALT/AST within the reference range (Rampon et al., 2020).

The limitation of the further therapeutic use of fomepizole is that the drug has to be administered as early as possible as the therapeutic window is limited to the drug metabolism phase (Akakpo et al., 2020).

We analyzed the effect of fomepizole in two type of studies for different primary outcomes in patients overdosed with paracetamol. A very low-certainty evidence suggests that a fomepizole treatment led to a significant decrease in fraction of ingested paracetamol excreted as total oxidative metabolites in 24-h urine compared to placebo (mean difference: 3.97%, 95% confidence interval: 2.31–5.63%, *p* = 0.003). Additionally, we summarized results from the three case studies describing eight patients treated with fomepizole after DILI was diagnosed. No control group was involved. Based on very low-certainty evidence the laboratory markers of hepatic function (ALT; AST; bilirubin, INR) were decreased in fomepizole-treated patients.

#### Livina

Livina is a polyherbal preparation consisting of 12 plants extracts: *Picrorhizha kurroa*, *Phyllanthus niruri*, *Andrographis paniculata*, *Cichorium invitybus*, *Tephrosia purpurea*, *Solanum dulcamara*, *Crenum aciaticum*, *Astonia seholanis*, *Holarrhave antidysentric*, *Tinospora cordifolia*, *Terminala chebula*, and *Asteracantha longifolia*. Its hepatoprotective effect was observed in ethanol-induced hepatic damage in rats, but the mechanism of hepatoprotection was not fully elucidated (Darbar et al., 2009). Livina significantly reduced elevated levels of ALT, AST, ALP, blood urea nitrogen and bilirubin after ethanol toxicity and prevented a significant increase in malondialdehyde level (*p* < 0.05), a marker of lipid peroxidation in rats.

Gulati et al. investigated a hepatoprotective effect of Livina in patients treated with antituberculosis drugs. In this single-blind placebo-controlled RCT involving 42 patients with tuberculosis the efficacy and safety of Livina were assessed. The compliance to treatment with antituberculosis drugs was also evaluated respecting to Livina therapy. Although the title indicates that Livina is given in prevention only, it is being tested as prevention and treatment. Livina was given 4 and 8 weeks after initiation of antituberculosis drug therapy. No data about the DILI causality assessment was available (Gulati et al., 2010).

A significant increase was observed for AST, ALT, ALP in placebo group comparing the 8 weeks’ levels to those at baseline measurement (ALT 52.5 ± 7.6 IU/L vs. 18.8 ± 3.7; AST 51.9 ± 10.5 IU/L vs. 24.4 ± 5.0; ALP 386.2 ± 29.3 U/L vs. 207.9 ± 20.1; *p* < 0.05) and this difference was not significant when comparing the Livina-treated group at 8-week time point to baseline. There was no direct comparison between investigated treatment. We found that the evidence is very uncertain about the effect of Livina on the reported outcomes.

#### Magnesium Isoglycyrrhizinate

Magnesium isoglycyrrhizinate is a magnesium salt of 18-α glycyrrhizic acid stereoisomer, refined from glycyrrhizic acid, which is extracted from the roots of herb *Glycyrrhiza glabra*. It is used in China in treatment of inflammatory liver diseases (Niu et al., 2021). Its mechanism of action in humans remains to be fully elucidated, however, scavenging of free radicals, prevention of the increase of serum transaminase, reduction of hepatocyte degeneration, necrosis and inflammatory cell infiltration might be included (Wang et al., 2019; Niu et al., 2021). Previous research has shown that MgIG has anti-inflammatory, antioxidant, and hepatoprotective activity in toxin-associated liver injury and hepatitis (Jiang et al., 2017a; Qu et al., 2017; Zou et al., 2018). Experimental studies in rodents demonstrated protective action against methotrexate, cyclophosphamide, paclitaxel, lipopolysaccharide, concanavalin A, ethanol, and free fatty acid (Cheng et al., 2009; Chen et al., 2014; Yang et al., 2016; Jiang et al., 2017a; Jiang et al., 2017b; Lu et al., 2017; Cao et al., 2019).

Wang et al. carried out a phase II, double-blind RCT in 174 DILI patients to assess the efficacy and safety of the MgIG, as compared to tiopronin, a standard therapy for liver injury in China. DILI was caused by different drugs (antituberculotic drugs, antitumor drugs, traditional Chinese medicine, antibiotics, cardiovascular drugs, anti-inflammatory drugs, hormones). Patients with a RUCAM score ≥6 were included in the study and were randomized into three groups: the low dose (100 mg MgIG, *N* = 59), the high dose (200 mg MgIG, *N* = 56), and the tiopronin group (200 mg tiopronin, *N* = 59). All substances were administered intravenously, once a day for 4 weeks. Patients were followed up once a week for a total of 4 weeks. The primary endpoint was a proportion of patients with serum ALT normalization at week 4. The association between drug treatment and ALT level at each investigated time point, changes in AST level at week 4, and proportion of patients experiencing adverse events in every group were secondary endpoints (Wang et al., 2019). Although the study provides evidence that MgIG is effective and safe treatment in patients with DILI, it poses certain limitations (e.g., short term follow-up, lack of full description of study design) which imply that results must be interpreted with caution.

The second study of MgIG that we aimed to include was a phase IV, single-blind RCT in 73 patients exploring the efficacy and safety of polyene phosphatidylcholine injection (930 mg once a day) in the treatment of acute DILI compared to MgIG injection (200 mg once a day) as the active comparator. Patients with a RUCAM score ≥6 were included in the study. This study was completed in China in 2020, but currently, there are no results available (NCT04595916, 2020).

The effect of MgIG 100 and 200 mg was evaluated in one published RCT comparing it to the effect of tiopronin. Both doses of MgIG likely result in a large increase in proportion of patients experienced ALT normalization (84.75%; 85.71%; *p* = 0.0111; *p* = 0.0087; moderate certainty evidence).

#### Picroliv

Picroliv, iridoid glycoside mixture of mainly picroside (6-O-trans cinnamoylcatalpol) and kutkoside (10-O-vanilloylcatalpol), is isolated from the roots and rhizomes of the endemic Himalayan plant Picrorhiza kurroa. An antioxidative, immunomodulating, and hepatoprotective, including antiviral, anticholestatic, and hepatoregenerating, activity has been related to this agent (Verma et al., 2009). The mechanism of the hepatoprotective effect includes antioxidant and membrane-stabilizing activities. Picroliv is able to inhibit the production of reactive oxygen species, neutralize free oxygen radicals, and stop lipid peroxidation (Picrorhiza, 2001; Verma et al., 2009). Animal studies showed picroliv could prevent or reverse the effects of liver toxins e.g. alcohol (Saraswat et al., 1999), paracetamol (Girish et al., 2009), rifampicin (Saraswat et al., 1997b), oxytetracycline (Saraswat et al., 1997a), carbon tetrachloride (Girish and Pradhan, 2012), galactosamine, and thioacetamide (Dwivedi et al., 1993).

The data investigating picroliv activity in humans has been related to one RCT. Based on the protocol published online in 2002 (Gaur and Bhosale, 2002), it was planned that two groups of patients treated with antituberculosis drugs (*N* = 260), i.e., one group treated with picroliv and another treated with placebo, would be evaluated for the primary endpoint, the number of patients who develop hepatotoxicity in each group, and secondary endpoint, safety assessment. Drug-induced liver injury was assessed according to the Councils for International Organizations of Medical Sciences based criteria. The results of this trial were published in abstract form only 11 years after the protocol as the incidence of antituberculosis drug-induced hepatotoxicity (Bhosale et al., 2013). The endpoints published in protocol and results published in the abstract markedly did not match. In the abstract, no data about picroliv treatment were described, and the observed incidence of hepatotoxicity was attributed to antituberculosis treatment only (Gaur and Bhosale, 2002; Bhosale et al., 2013).

The results of this study remarkably differ from those prespecified in the protocol. The primary outcome described in the protocol, that was the number of patients who develop hepatotoxicity during TBC treatment and concomitantly treated with picroliv was of interest for this review. As this outcome was not reported in the published abstract, we were not able to assess the certainty of the evidence for this outcome. Limitations of this study are significant and conclusions should be very cautiously interpreted. We included this study in analyses aiming to avoid the mistake of selective outcome reporting. This study also contributed to the whole certainty of the evidence and we tried to give as much insight as possible in this field of research.

#### Plasma Exchange

Plasma exchange, PE or TPE, is a therapeutic intervention for the first time used in humans in the 1950s to treat hyperviscosity due to Waldenstrom macroglobulinaemia (Skoog and Adams, 1959). It refers to an extracorporeal procedure when the patient’s plasma is separated from whole blood and removed, while the cellular blood components (red blood cells, granulocytes, lymphocytes, monocytes, and platelets) are returned to the patient. Plasma components that are removed from the patient’s circulations include fibrinogen, complement, immunoglobulins, and cryoglobulins, including antibodies, lipids (e.g., cholesterol), enzymes (e.g., ALP, creatinine phosphokinase), and small molecules (e.g., uric acid, potassium, toxins). Opposite reports exist regarding the effect of TPE on the concentration of cytokines and adhesion molecules (Reeves and Winters, 2014). Although a clear indication for TPE is established for 84 diseases and 157 indications (Padmanabhan et al., 2019), reports on TPE used in DILI are scarce. In four case reports, one case series and two letters to the editor 19 patients with DILI were successfully treated with TPE (Aydemir et al., 2005; Bilgir et al., 2013; Liu et al., 2013; Göpel et al., 2016; Philips et al., 2017; Riveiro-Barciela et al., 2019; Rong et al., 2020). In two studies, RUCAM tool was used to assess the causality between the suspected drug and liver injury (Philips et al., 2017; Rong et al., 2020). Persistent liver injury was defined as a serum AST or ALT level >1.5 × ULN or an ALP level > ULN at 6 months after DILI onset by Rong et al. (2020). All included studies descriptively assess the course of drug hepatotoxicity related to investigated therapeutic intervention.

Sachan et al. (2017) and Jothimani et al. (2018) published a retrospective study including 10 patients and 26 TPE procedures. A described outcomes included improvement in aminotransferases, direct bilirubin and INR. A DILI causality assessment was based on recent onset of jaundice, history of intake of hepatotoxic drugs, and exclusion of possibilities of other etiologies of liver injury.

The efficacy and safety of TPE compared to SMT in 30 patients of severe DILI were investigated in a RCT (Sinha et al., 2020). In this study, DILI was assessed based on history and liver biopsy (*n* = 24, 80%) and graded for the severity as by DILIN score. Fifty-three TPE procedures were performed. The outcomes were: bilirubin and bile acid levels, INR, and IL-6/TNF-α/IL-1β at day 7 (Sinha et al., 2020).

We also found articles describing the efficacy of TPE in DILI patients, but the participant groups were not coherent regarding etiology and included different causes of liver injury (Hung et al., 2004; Akdogan et al., 2006; Li et al., 2016) and those articles we did not describe in Table 2 as we considered them less relevant for this review. Exceptionally, we present here the results of two studies that included different causes of liver injury, regardless of underlying etiology. In the open-label RCT Larsen et al. investigated the therapeutic effect of PE in ALF. The authors showed a higher survival rate in patients treated with PE, irrespective of etiologies of ALF. Therapeutic PE served as a “buying time” procedure, bridging the gap of ALF to transplantation (Larsen et al., 2016). Additionally, a systematic review investigating the efficacy of PE in acute and acute-on-chronic liver failure found that this procedure improves survival at 30-and 90-day in non-transplanted patients (Tan E. X. et al., 2020).

We evaluated the efficacy of PE in three types of studies. Two of those were published in abstract form only and third included case reports, series and letters to the editors of 19 patients in total. The primary outcomes vary substantially within included studies: laboratory parameters and clinical signs and symptoms of liver function (e.g., AST, ALT, bilirubin, INR; <20% reduction in serum bilirubin from baseline after 3 TPE sessions or reduction in serum bilirubin by < 5 mg and INR <1.5). No control treatment was involved. A positive effect of PE to outcomes reported in all included studies was descriptively demonstrated. We found that TPE may reduce or have little to no effect on reported outcomes based on very uncertain evidence.

#### Radix Paeoniae Rubra

Radix Paeoniae Rubra is a traditional herbal Chinese medicine widely used in clinical practice. It poses immunomodulatory, antioxidant, hepatoprotective, cardioprotective, neuroprotective, sedative, antidepressant, anticonvulsant, analgesic, and anti-tumor effects (Tan Y. Q. et al., 2020; Zhou et al., 2020). The hepatoprotective effects are attributed to paeoniflorin, ethyl linoleate, and ethyl palmitate and related to inhibition of inflammatory processes, the scavenge of free radicals and the limitation of destructive effects produced by oxidative stress on the liver tissue (Zhou et al., 2020).

The efficacy and safety of RPR in the treatment of 14 severe DILI patients with intractable jaundice caused by antibiotics, paracetamol and alternative medications were investigated in the study conducted in China and published in 2017. Included patients failed to respond to the withdrawal of the suspected drug and were previously unsuccessfully treated with UDCA, corticosteroids, and PE. No data about the DILI causality assessment was available. Biochemical parameters of liver function (AST, ALT, TBL, direct bilirubin, total bile acid, albumin, cholinesterase), symptoms of liver injury (jaundice and fatigue), Child-Pugh and MELD scores comparing the same patient before and after RPR treatment and the need for liver transplantation were assessed as outcome measures. No control treatment was involved. This study included only severe DILI patients who failed to respond to previous therapy and follow-up time was appropriate (Jing et al., 2017). Simultaneously, in 2017 European Medicine Agency published Assessment report on RPR, namely paeoniflorin, concluding that oral bioavailability of this natural compound is very low (3–4%) and no adequate information exists for this compound to make any conclusion in human posology.

The evidence is very uncertain about the effect of RPR that was described to significantly decrease laboratory parameters when compared to baseline values (ALT 101 ± 91 vs. 38 ± 35 U/L, AST 113 ± 77 vs. 49 ± 29 U/L, TBL 20.6 ± 6.1 vs. 4.9 ± 8.2 mg/dayL, direct bilirubin 15.2 ± 5.3 vs. 3.2 ± 6.1 mg/dayL, total bile acid 282 ± 134 vs. 50 ± 74 mol/L; all *p* < 0.05). Additionally, RPR may reduce jaundice (100 vs. 21%; *p* < 0.001) and fatigue (86 vs. 29%; *p* = 0.006), but the evidence is very uncertain.

#### S-Adenosylmethionine

S-adenosylmethionine, an endogenous substance derived from methionine in the liver, is a precursor of GSH and has an important role in the prevention of oxidative stress. S-adenosylmethionine is essential as a donor of methyl group for transmethylation of phospholipids, being crucial in maintaining the structure and function of cell membranes. It has been suggested that due to its depressed biosynthesis in chronic liver diseases, SAMe supplementation attenuates liver injury through facilitating GSH synthesis, the major endogenous hepatoprotective agents, decreasing inflammation by inhibition of TNF-α, and the regulation of IL-10 production (Mora et al., 2018). The administration of SAMe has been evaluated in the prevention and in treatment of a variety of liver injuries such as chronic hepatitis, alcoholic hepatitis, and paracetamol-related liver damage (Vincenzi et al., 2018). Considering the fact that SAMe enhances synthesis of hepatoprotective GSH, it has been evaluated in the prevention and treatment of chemotherapy-induced hepatotoxicity.

The effect of SAMe supplementation, 400 mg orally TID, in 50 patients diagnosed with metastatic colorectal cancer (CRC) and chemotherapy-induced liver toxicity was evaluated in a prospective study (Santini et al., 2003). The outcome measures included the proportion of patients achieved a reduction of AST, ALT, and lactate dehydrogenase (LDH) after 2 weeks and the number of chemotherapy cycles delays or dose reductions due to transaminases elevation. Two observational studies described the protective and therapeutic effects of SAMe in cancer chemotherapy-induced liver toxicity. For chemotherapy-induced hepatotoxicity, the first increase of one or both transaminases of at least 2.5 normal values was considered. Liver toxicity was assessed in every therapy cycle according to NCI-CTCAE, version 3, and chemotherapy course delays, discontinuations, and dose reductions due to liver toxicity were analyzed. Patients with CRC (*N* = 105) treated with 12 cycles of adjuvant FOLFOX regimen (oxaliplatin, folinic acid, 5-fluorouracil (5-FU) considered as hepatotoxic causative agents) and SAMe 400 mg TID concomitantly were compared to the patients who have been treated with no supplement treatment in a retrospective study (Vincenzi et al., 2011). The effect of SAMe was investigated comparing the level of AST, ALT, LDH, TBL, ALP, gamma-glutamyl transferase (GGT) and the grade of liver toxicity (range 0–4; lower grade, less toxicity). The next study conducted by the same authors showed the effectiveness of the SAMe in the same dosage on 78 patients affected by metastatic CRC and treated with bevacizumab plus XELOX regimen (capecitabine and oxaliplatin plus oxaliplatin, capecitabine, considered as hepatotoxic causative agents) (Vincenzi et al., 2012).

The effect of SAMe was compared to placebo in patients with cancer treated with chemotherapeutic drugs based on three studies. The primary outcomes were laboratory markers of liver function: AST, ALT, LDH, TBL, GGT, and ALP. In each study, a significant positive effect was observed for SAMe treatment on the prespecified outcomes (*p* at least ≤0.04). It was not possible to summarize results from the all three studies as partial data were reported (only means, without standard deviation in two studies and one study was available only in abstract form descriptively reported outcomes). S-adenosylmethionine may reduce or have little to no effect on prespecified outcomes, but the evidence is very uncertain.

## Discussion

Drug-induced liver injury is an uncommon but potentially severe hepatic disorder. It becomes an increasing public health burden and remains a relatively orphan disorder in terms of effective treatment options. To our knowledge, this is the first systematic review that summarizes novel agents currently under clinical investigation or repurposed, with presumed therapeutic effectiveness in DILI in humans. In general, therapeutic approach in DILI starts with physician suspicion of the association of certain drug(s) with various phenotypes and severity of DILIs (clinical manifestation and laboratory abnormalities), exclusion of alternative etiologies that could lead to similar liver injuries, causality assessment, making decisions on further treatment in terms of risk of continuance or discontinuation of the implicated drug in combination with supportive treatment if necessary, and/or re-challenges to ascertain tolerance or drug adaptation (Stine and Lewis, 2016; Hoofnagle and Björnsson, 2019). It has been confirmed that most of the patients including those with drug-induced jaundice recover clinically and biochemically. Particularly, patients with DILI associated with jaundice and coagulopathy should be closely monitored with monitoring of liver function tests and INR >1.5 considering hospitalization (Björnsson, 2021).

The evidence-based guided and well-established treatment exists for the few specific DILI indications, depending on etiology. In addition to NAC, the approved antidote for the paracetamol-induced liver injury, N-acetylcysteine amide (NACA) has been investigated in recent years (Tobwala et al., 2015; Khayyat et al., 2016; Chiew et al., 2018). N-acetylcysteine amide also acts as a GSH precursor, but its liposolubility and bioavailability are higher requiring lower doses with potentially fewer adverse effects (Khayyat et al., 2016). We found only two studies demonstrated the better effect of NACA compared to NAC: *in vitro* in human hepatoma HepaRG cells (Tobwala et al., 2015) and *in vivo* in mice (Khayyat et al., 2016), but none has been published in humans yet. Although many reports have been published about the positive effect of NAC treatment in the non-paracetamol-induced liver injury, a recent Cochrane review updated this indication and concluded that current evidence does not support the guideline suggestion to use NAC in adults with non-paracetamol-related ALF (Lee et al., 2009; Singh et al., 2013; Hu et al., 2015; Borlak et al., 2018; European Association for the Study of the Liver, 2019; Siu et al., 2020; Devarbhavi et al., 2021a).

The beneficial effects of corticosteroids for immune-mediated DILI was reported in case reports and observational human studies for antimicrobials (nitrofurantoin, minocycline), non-steroidal anti-inflammatory drugs (diclofenac, metamizole), TNF-α inhibitors, imatinib, etc. (Björnsson et al., 2010; Efe et al., 2010; Chalasani et al., 2015; Sawada et al., 2019; Weber et al., 2019). A RCT data (protocol) investigating the effect of corticosteroids on the chronic recurrent DILI was published online (Zhengsheng, 2016). The study is completed, the results have not been posted yet, but the authors have implicated that corticosteroids showed a positive effect in this trial (Wang et al., 2021). However, a recent systematic review pointed out the possibility of avoiding corticosteroids treatment in patients with DILI induced by immune checkpoints inhibitors (Peeraphatdit et al., 2020), and that not all immune-mediated DILIs, particularly those associated with prolonged jaundice, respond to corticosteroid administration (Hu et al., 2015).

The most recent guidelines by Asian and European expert groups were consistent in their positive recommendation for cholestyramine treatment for leflunomide-induced liver injury and carnitine treatment for valproate-induced liver injury (European Association for the Study of the Liver, 2019; Devarbhavi et al., 2021a). Both recommendations are based on case reports and observational studies, as RCTs in this field are lacking (Mock and Schwetschenau, 2012; Laub et al., 2016; Gandam Venkata et al., 2021). Additionally, studies in humans investigating the efficacy of colestipol hydrochloride and colesevelam hydrochloride to accelerated elimination of teriflunomide in healthy volunteers were published in 2017 (Lunven et al., 2017; Robertson et al., 2017).

The efficacy of UDCA in the treatment of cholestatic DILI still has not been proven in RCTs (Sundaram and Björnsson, 2017; Cabrera et al., 2019). American and European expert panels do not recommend its use (European Association for the Study of the Liver, 20192; Chalasani et al., 2021).

In addition to nonspecific liver support treatment strategies (e.g. gastric lavage, activated charcoal), a TPE and molecular adsorbent recirculating system (MARS) could have a potentially important, even crucial role in DILI treatment (Spiller et al., 2006; Stine and Lewis, 2016). The role of TPE and MARS should be to serve as a “buying time” procedure, replacing liver dysfunction for a short time and/or bridging the gap between liver dysfunction to transplantation. Up to date, opposite results were shown considering the improvement in morbidity and mortality in ALF related to drug toxicity treated with TPE and MARS (Wittebole and Hantson, 2011; Donati et al., 2014; Díaz et al., 2016; Larsen et al., 2016; Eapen et al., 2018).

Additionally, recent advances in the bioscience and stem cell field have developed various *in vitro* models, such as organoids, organ-on designed for reproducing human tissues and organs, being useful in researching novel treatments (Jin et al., 2021; Kulkeaw et al., 2021; Shinozawa et al., 2021). Therapy based on the mesenchymal stem cells (MSCs), is an innovative current method commonly preferred due to their advantages as broad therapeutic potential and easy accessibility. Mesenchymal stem cells proved to have anti-inflammatory, immunomodulatory, and antioxidant properties in various pathological states (Luan et al., 2021). Based on self-renewal potential and differentiation ability in hepatocyte-like cells researches using animal models of carbon tetrachloride and α-galactosylceramide toxic liver injuries have proved the favorable effect of MSCs on function and liver damage (Alfaifi et al., 2018; Wang et al., 2018). Mesenchymal stem cells based therapy has been advanced as a substitute for liver transplantation, especially in alcoholic and viral cirrhosis, but has not yet surpassed the early phases of clinical studies (Lee et al., 2021). To date, no clinical trials have been conducted to evaluate the effects of MSCs in DILI. The possible reasons could be the sudden onset of liver damage, severe evolution, and increased mortality rate, all of which require more conventional and conservative therapeutic methods (Zhou et al., 2020).

### Summary of Main Findings

Although many drugs considered as therapeutic options for DILI have been investigated in animal studies, this review focuses on the drugs that showed efficacy in clinical practice.

We found only for the outcomes of MgIG a moderate certainty of the evidence and for the outcomes of bicyclol treatment a low certainty of the evidence. For remaining eight interventions, the certainty of the evidence for primary outcomes was assessed as very low, that means we are very uncertain in any estimate of effect.

Magnesium isoglycyrrhizinate is an agent that could be listed as a therapeutic option for DILI in the near future worldwide. It demonstrated efficacy in DILI treatment, according to phase II RCT (Tang et al., 2012; Wang et al., 2019). Similarly, in a phase III RCT published in Chinese only, a significant ALT normalization rates in patients with chemotherapeutic acute DILI treated with MgIG were observed, as compared to tiopronin (Tang et al., 2012).

Evidence for the efficacy of bicyclol in the treatment of DILI is still scarce, although its marked hepatoprotective effects have been reported in clinical trials (European Association for the Study of the Liver, 2019, Chalasani et al., 2021). In the analysis of RCTs with bicyclol, Niu et al. (2021), demonstrated reduced incidence and significant normalization rates of laboratory abnormalities in the treatment or prevention of DILI. In a Chinese pharmacoeconomic study four hepatoprotective drugs (bicyclol, tiopronin, reduced glutathione, and diammonium glycyrrhizinate) in DILI treatment were analyzed, and the authors concluded that bicyclol had the greatest efficacy and safety, as well as favorable cost-effectiveness ratio (Huang and Wang, 2014).

Calmangafodipir has a rational potential to be an effective drug in DILI treatment. It has been positively rated in phase I clinical trial and the industrial developer has intended to conduct a pivotal phase IIb/III trial for liver damage associated with paracetamol poisoning in the second half of 2021 (Morrison et al., 2019; Insight, 2021).

Cytisin amidophosphate, a drug only approved in Kazakhstan for clinical trials, may be used to treat DILI. Nonetheless, that only one RCT with cytisin amidophosphate as a therapeutic agent for acute toxic hepatitis induced by drugs has been reported, findings by Zhumadilov et al. (2012), showed clinical improvements and normalization in aminotransferase and bilirubin levels.

S-adenosylmethionine administration has shown to be effective in the established cholestasis and is associated with the improvement of symptoms in chronic hepatitis, pregnancy-induced liver injury, alcoholic hepatitis, and paracetamol-induced liver injury (Anstee and Day, 2012). Although several retrospective studies (Santini et al., 2003; Vincenzi et al., 2011; Vincenzi et al., 2012) investigated empirical administration of this drug in both prevention and treatment of chemotherapy-induced liver injury and showed obvious improvement in clinical course and prognosis, large prospective and randomized studies are still needed to determine its real clinical benefits in DILI treatment.

Fomepizole proved to be effective in paracetamol or alcohol overdose due to its mechanism of action, but the therapeutic window is very narrow and restricted to the very early metabolism phase (Jaeschke et al., 2020). However, because of action on the different therapeutic targets in comparison to NAC as a standard of care, it could provide additional benefits in the treatment of DILI triggered with other drugs than paracetamol, but for early presenting patients and as an adjunctive treatment.

Herbal drugs and plants, including complementary and alternative medical supplies, are historically widely used in Chinese and Indian medicine practice. However, many limitations are related to studies assessing the efficacy of those agents. Articles published on their hepatoprotective effect in DILI are frequently structured as non-RCT (Jing et al., 2017), limited in sample size [e.g., small sample size, not representative sample (Gulati et al., 2010)] and/or introducing a substantial reporting bias [results have not been published yet or differ significantly from the published protocol (Gaur and Bhosale, 2002)], results were published exclusively and only in abstract form (Jing et al., 2017). Natural compounds often pose a challenging pharmacology profile (e.g., picroside and paeoniflorin). Their pharmacokinetic and pharmacodynamics properties frequently limit or disable interpolating results from animals to humans (Upadhyay et al., 2016). Finally, many herbal agents, solely or as mixed preparation, are at risk to cause DILI (Devarbhavi et al., 2021b; Larson, 2021). Regarding the fact that they are nowadays available worldwide for the different spectra of diagnoses and that many people think that they are harmless, clinicians could reasonably suspect herbal drugs and plants as an offending in the setting of DILI patients. Clinical studies in humans in this field, especially RCTs, are lacking. In the review on the commonly used 27 pure natural compounds, Potočnjak and Domitrović analyzed the results of mostly animal model studies, but it could serve as a respectful basis and cornerstone to plan clinical studies in humans (Domitrović and Potočnjak, 2016).

### Limitations

This review represents an up-to-date, comprehensive, and evidence-based critical assessment of the potentially novel agents or repurposed drugs considered for DILI treatment in clinical practice, including investigational and practical use, worldwide. However, a few limitations should be highlighted. Since there are not many high-quality clinical trials, such as RCTs, we decided to include all types of clinical research in humans, i.e., observational (retrospective and prospective studies, case reports, case series), RCTs as well as studies published in abstracts or poster forms only. This fact introduced a very wide observational difference between analyzed participants, outcomes and intervention. The national or local criteria of acute toxic hepatitis induced by drugs, and observation of clinical courses in DILI diagnosis and the treatment rather than standard, uniform DILI definition and causality assessment represent a significant limitation of included studies. Consequently, this limited us to draw a uniform, homogenous conclusions rather than descriptively reported summarized outcomes. This fact contributes to higher heterogeneity between included studies and decreases the methodological quality of the overall body of the evidence, but simultaneously provides a more comprehensive insight into this area of research and achievements to date. Aiming not to narrow the scope of published evidence, we included protocols and studies based on them or only published protocols, if there was no study published. We reported those partial contributions above. Furthermore, only publications in English were evaluated and this may represent a limitation. Although Chinese, Russian and Indian scientific contributions were included if their manuscripts were available in English, we certainly omitted a bunch of papers that have been published in the original languages only.

## Conclusion

The DILI has been one of the most severe liver disorders necessitating the development of safer drugs. However, scarce evidence-based therapeutic options exist for DILI. There is no topgallant medicine to treat DILI irrespective of etiology. In the case of non-self-limited liver damage and drug-induced ALF, liver transplantation stands as a golden standard treatment. Despite the detailed mechanism of action of the promising drug candidates in DILI treatment, their real efficacy and safety undoubtedly need to be confirmed in future RCTs. Considering the low number of RCT trials, poor description of methodological design, heterogeneous DILI diagnostic criteria, rare use of RUCAM scale, short-term follow-up, and lack of clinically meaningful endpoints, the certainty of the evidence is very low and conclusions based on presented evidence should be interpreted cautiously.

## Glossary

ALF: acute liver failure
ALP: alkaline phosphatase
ALT: alanine aminotransferase
AST: aspartate aminotransferase
CENTRAL: The Cochrane Central Register of Controlled Trials
CMF regimen: Cyclophosphamide, Methotrexate, 5-Fluorouracil
CRC: colorectal cancer
DILI: drug-induced liver injury
DILIN: Drug-Induced Liver Injury Network
DRESS: drug-related eosinophilic systemic syndrome
FOLFOX regimen: Oxaliplatin, FA, 5-FU Leucovorin Calcium (Folinic Acid), Fluorouracil, and Oxaliplatin
FOLFRI regimen: 5-Fluorouracil, Irinotecan
GGT: gamma-glutamyl transferase
GRADE: Grading of Recommendations Assessment, Development and Evaluation
GSH: glutathione
ICTRP: International Clinical Trials Registry Platform
iDILI: idiosyncratic drug-induced liver injury
INR: international normalized ratio
LDH: lactate dehydrogenase
MARS: molecular adsorbent recirculating system
MgIG: magnesium isoglycyrrhizinate
MELD: Model for End-Stage Liver Disease
MSCs: mesenchymal stem cells
NCI-CTCAE: National Cancer Institute—The NCI Common Terminology Criteria for Adverse Events
NAC: N-acetylcysteine
NACA: N-acetylcysteine amide
NAPQI: N-acetyl-p-benzoquinone imine
NSAIDs: nonsteroidal anti-inflammatory drugs
PD-1: programmed cell death protein
PRISMA: the Preferred Reporting Items for Systematic Reviews and Meta-Analyses
RCT: randomized controlled trial
RPR: Radix Paeoniae Rubra
RUCAM: Roussel Uclaf Causality Assessment Method
(S)AES: (serious) adverse events
SAMe: S-adenosylmethionine
SMT: standard medical treatment
TBL: total bilirubin
TID: three times a day
TNF-α: Tumor Necrosis Factor-α
(T)PE: (therapeutic) plasma exchange
UDCA: ursodeoxycholic acid
ULN: upper limit of normality
WHO: World Health Organization
XELOX regimen: Capecitabine (Xeloda) and Oxaliplatin, Oxaliplatin, Capecitabine
