# Supplementary material for: Novel Therapies for the Treatment of Drug-Induced Liver Injury: A Systematic Review
Source: Front Pharmacol. 2022 Feb 2;12:785790. doi: 10.3389/fphar.2021.785790 (PMC8847672; doi:10.3389/fphar.2021.785790)
Supplement: Supplementary file 1 [file Table1.docx]

Supplementary Table 1: Risk of bias across included studies with detailed explanations

| Study/ agent | Risk of bias domain | | | | |
| --- | --- | --- | --- | --- | --- |
|  | **Selection bias** (random sequence generation and allocation concealment) | **Performance and detection bias** (blinding of participants, personnel and outcome assessment) | **Attrition bias** (incomplete outcome data) | **Reporting bias** (selective reporting) | **Other bias** |
| Wu et al., 2017  Bicyclol | | | | | |
| Judgment (low/unclear/high) | LOW | UNCLEAR | LOW | LOW | UNCLEAR |
| Support for judgments | Randomized, parallel-design controlled trial; the random number table was generated by SPSS 22.0 statistical analysis software. | Method not described. | Dropouts were adequately balanced and minimal (157/168 patients completed the study). | All prespecified outcomes were adequately reported. | No data on conflict of interest and sponsorship. Sample size assessed before the study enrolment. |
| Morrison et al., 2019; The POP Trial Investigators. and Dear, 2019  Calmangafodipir | | | | | |
| Judgment (low/unclear/high) | LOW | HIGH | LOW | LOW | HIGH |
| Support for judgments | The allocation sequence for each dosing cohort was created by a programmer using computer-generated random numbers. The randomisation list was held centrally in order to conceal treatment allocations until these were implemented via the secure web-based randomisation system. | There was no blinding of participants or emergency department staff. The statistical analysis plan was written blinded to the treatment allocations. | There were no withdrawals. | All prespecified outcomes were adequately reported. | Funded by the Sponsor, PledPharma AB,tockholm. Protocol was published and available online. Sample size did not *apriori* assessed. |
| Zhumadilov et al., 2012  Cytaphat | | | | | |
| Judgment (low/unclear/high) | UNCLEAR | UNCLEAR | LOW | LOW | UNCLEAR |
| Support for judgments | Method not described. No information about the strata of differences in baseline demographic and clinical characteristics. | Method not described. | 3.52% (5/142) patients drop out from the study and reasons were adequately described. | All outcomes stated in the Method section were reported. | No data on conflict of interest and sponsorship.  Sample size was not assessed before the study enrolment. |
| Kang et al, 2020  Fomepizole | | | | | |
| Judgment (low/unclear/high) | LOW/UNCLEAR | UNCLEAR | LOW | LOW | HIGH |
| Support for judgments | Randomized by blind draw from an envelope to start with one of two treatments, but allocation was not concealed due to cross over study design. | Method not described. | 16.67% (1/6) patients withdrew due to reason adequately described. | All outcomes stated in the Method section were reported. | Each participant received a $200 gift card for participating. |
| Zell-Kanter et al. 2013, Shah 2020., Rampon et al, 2020  Fomepizole | | | | | |
| Judgment llow/unclear/high) | HIGH | HIGH | UNCLEAR | UNCLEAR | UNCLEAR |
| Support for judgments | Not randomized. | Not double blinded. | Descriptive report of clinical cases. | Descriptive report of clinical cases. | Case reports and series (6 cases). |
| Gulati et al., 2010  Livina | | | | | |
| Judgment (low/unclear/high) | LOW | HIGH | UNCLEAR | LOW | UNCLEAR |
| Support for judgments | Computer generated randomization chart. | Single blind study. | 6/48 patients withdrew from the study, reasons not described. | All outcomes stated in the Method section were reported. | No data on conflict of interest and sponsorship. Sample size was not assessed before the study enrolment. |
| Wang et al., 2019  Magnesium Isoglycyrrhizinate | | | | | |
| Judgment (low/unclear/high) | UNCLEAR | UNCLEAR | LOW | LOW | HIGH |
| Support for judgments | Method not described. | Method not described. | Analyses performed as ITT. All dropouts (19/174) were adequately reported and reasons described. | All outcomes stated in the Method section were reported. | Sponsored by Chia Tai Tianqing Pharmaceutical Group Co., Ltd. Sites involved in the study were also supported by the Major Project of National Twelfth Five Plan and the Major Project of National Thirteenth Five Plan. |
| Gaur and Bhosale, 2002; Bhosale et al., 2013  Picroliv | | | | | |
| Judgment (low/unclear/high) | UNCLEAR | UNCLEAR | UNCLEAR | HIGH | UNCLEAR |
| Support for judgments | Method not described. | Method not described. | Not described. | The prespecified outcomes remarkable differ from those published in abstract form only. | Conflict of interest and sponsorship data not reported. |
| Jothimani et al., 2018; Sachan et al., 2017  Plasma exchange | | | | | |
| Judgment (low/unclear/high) | HIGH | HIGH | UNCLEAR | HIGH | UNCLEAR |
| Support for judgments | Not randomized. | Not double blinded. | Descriptive report of clinical cases. | Descriptive report of clinical cases in abstract form only. | Conflict of interest reported. Sponsorship data not reported. |
| Aydemir et al., 2005; Bilgir et al., 2013; Liu et al., 2013; Göpel et al., 2016; Philips et al., 2017; Riveiro-Barciela et al., 2019; Rong et al., 2020  Plasma exchange | | | | | |
| Judgment (low/unclear/high) | HIGH | HIGH | UNCLEAR | HIGH | UNCLEAR |
| Support for judgments | Not randomized. | Not blinded. | Descriptive report of clinical cases. | Descriptive report of clinical cases. | Case reports and series (19 cases). |
| Sinha et al., 2020  Plasma exchange | | | | | |
| Judgment (low/unclear/high) | HIGH | HIGH | LOW | UNCLEAR | UNCLEAR |
| Support for judgments | Not randomized. | Not double-blind. | No withdrawals reported, although it was a descriptive study. | Descriptive report in abstract form only. It seems that prespecified endpoint described in Methods have not been reported in Results. | Conflict of interest, sponsorship data not reported. |
| Jing et al., 2017  Radix Paeoniae Rubra | | | | | |
| Judgment (low/unclear/high) | HIGH | HIGH | UNCLEAR | UNCLEAR | UNCLEAR |
| Support for judgments | Not randomized. | Not blinded. | Descriptive report of clinical cases. | Descriptive report in abstract form only. | Conflict of interest and sponsorship data not reported. |
| Santini et al., 2003, Vincenzi et al., 2011, Vincenzi et al., 2012  S-adenosylmethionine | | | | | |
| Judgment (low/unclear/high) | HIGH | HIGH | UNCLEAR | LOW | LOW/UNCLEAR |
| Support for judgments | Not randomized. | Not blinded. | Not described. | It seems that all outcomes stated in Methods were reported. | The authors declare no conflict of interest and no sponsors for one study. |
